# The Book World of Medicine and Science

**Published:** 1901-12-28

**Authors:** 


### The Book World of Medicine and Science.

Aids to Obstetrics. By S. Nall, M.B., D.P.H. Sixth edition. Students' Aids Series. (Baillière, Tindall and Cox. 8vo., pp. 140. Price 2s. 6d.)

A reviewer can find little fault with any book which has reached six editions. The author admits frankly that this volume is written for "cram" purposes, and we must accept it as such. Dr. Nall is careful to specify that no book of this kind will get any student through an examination, even the most elementary, without other reading. This being understood, there can be nothing but praise for this little manual. It contains everything essential to a manual on obstetrics in a very condensed form, and a due proportion of importance is assigned to those essentials. A well-written "primer" such as this arranges the perspective of the examinee's mind before examination, and teaches him that the small difficulties of elementary midwifery (such as passing a catheter) are not the points on which the examiner will lay stress, nor the troubles which will tax his knowledge in later practice. The requirements of space, probably enforced by the publishers, have had in one aspect a good result; they have prevented the compilation of tabular arrangements of conditions, methods of treatment, and other comparable notions, a method we believe to be radically wrong in training the memory, but one which has of late years established an unfortunate hold on smaller works of obstetrics and gynæcology. The section on deformity of the pelvis is particularly well written, and the impress of Matthew Duncan's personal teaching on the author may perhaps be traced therein. A few points in the book are open to criticism: more stress should have been laid on the immediate suture of all cases of ruptured perinæum, except the most minute, and something should be said on the choice of material of those sutures. More accurate instructions as to the application of the Faradaic current in cases of post-partum hæmorrhage, should be offered than are presented on p. 95. It is not always safe, moreover, to go poking about with an ordinary uterine sound in cases of suspected inversion of the uterus.

Menstruation and its Disorders. By Arthur E. Giles M.D., D.Sc., F.R.C.S., M.R.C.P. (London: Baillière, Tindall and Cox. 1901. Price 2s. 6d. net.)

There is a great deal in this book that is very interesting; indeed we do not know where else one could find in so small a compass so careful and so definite a description of the matter dealt with as is given in this short monograph. After a slight historical review of the subject, Dr. Giles describes the anatomical and physiological characters of menstruation and its relation to the "heat" or "œstrus" of animals. He shows that what is termed "heat" in animals must be divided into two stages, one in which the generative organs of the female show signs of special activity with some discharge of mucus or blood from the vagina, and the other a period varying from one to several days in duration, during which alone the female is capable of impregnation and will receive the male. The first of these may be called the pro-œstrum and the last the œstrus; and Dr. Giles takes the view that menstruation in women and in monkeys is homologous with the pro-œstrum in the lower animals. Thus menstruation may be defined, and is to be regarded as a periodic uterine preparation for pregnancy; and although it has to be admitted that there is a difference, in that ovulation always coincides with œstrus in animals, while it does not of necessity coincide with menstruation in women, it yet may be taken as true that the use of the degeneration and superficial stripping of the uterine mucous membrane which occurs at menstruation is the preparation of a surface on which the fertilised ovum may become ingrafted. That a difficulty in accepting this theory arises from the fact that impregnation is possible even during the week preceding menstruation is evident. For the way the author gets over this difficulty we must refer to the book itself, although we may say in passing that we are not entirely convinced that his explanation covers the whole ground. The various disorders of menstruation and the effects which other diseases are liable to have upon the process are well described both in regard to symptomatology and treatment. It is a book well worth reading.

The Evolution of Sex. By Professors Patrick Geddes and J. Arthur Thomson. The Contemporary Science Series. Illustrated. Revised edition. 8vo. (London Walter Scott. 1901. Price 6s.)

Like several of this series the volume before us suffers from an endeavour to squeeze a very wide and technical subject into small limits. The authors may, however, be complimented on the result they have achieved in so difficult a task. They begin with a brief sketch of sexual selection and secondary sexual attributes, culled avowedly from Darwin, and rounded off with a good criticism of the original postulates of Wallace and Darwin, and the further modifications of their following, particularly Brooks. The various factors which are believed to determine sex in offspring are then considered at full length, and the obvious and necessary importance attached to the effects of diet will recall the lay reader's sympathies to a lately discredited savant in Vienna. A short sketch of the multiform varieties of sexual organs in the invertebrata, is followed by an excellent chapter on Hermaphroditism in lower animals. These follow an excellent summary of the history of Embryology, beginning with Harvey's theories, and ending with the transcendental embryologists of the last twenty years, Haekel, Brooks, Nussbaum, and others, and last but not least Weismann. Any reader, not a trained biologist, who seeks an introduction to this difficult branch of biology, may be well advised to begin with this little volume. First, the index is good; next, the bibliographical summary at the end of each chapter is good; but particularly the historical sketches are good. In Chapter IX. the history of the recognition of the spermatozoon, and in Chapter X. the account of the growth of opinion on the differences between male and female from Drelincourt to Brooks, and in Book III. the summary of the processes of fertilisation are fine examples of the authors' claim to do no more than present a general survey of the essential phenomena of sex and reproduction. But to the careful reader they do present also an important theory, that the essential physiological difference between male and female organisms consists in the greater tendency of the female cells to build up and store living matter, that of the male cells to destroy such built-up material by an intenser activity; in the technical language of modern physiology the female is anabolic, the male is katabolic; and the most pronounced period of distinction between the sexes is seen when the sexes are represented by the ovum and the spermatozoon. The definite assertion of this principle has with a too great modesty been relegated to the prefaces, whereas, the authors might, in the chapter on the Reproductive Factor in Evolution, have pointed out that the tendency of the civilised woman to assume the katabolic physiological characters of the male will result quickly in the destruction of the anabolic characters of the female necessary to reproduction, quite apart from the artificial checks of Neo-malthusianism. The discussion on Neo-malthusianism in Chapter XX. is hardly germane to the general subject. The value of such comments in cheap text-books is doubtful, and most medical experts feel this. The authors accuse the medical profession of pusillanimity in approaching the subject, but fully-expressed views can be found in most authoritative works on gynæcology. The illustrations are mostly excellent, the index is sufficiently full, but several of the diagrams are obscure and hardly required.

